# Common Sleep Disorders in Patients With Chronic Kidney Disease: A Systematic Review on What They Are and How We Should Treat Them

**DOI:** 10.7759/cureus.44009

**Published:** 2023-08-23

**Authors:** Gershon G Davydov, Hiba Nashat, Sally Ghali, Shadin Afifi, Vineet Suryadevara, Yaman Habab, Alana Hutcheson, Binay K Panjiyar, Tuheen Sankar Nath

**Affiliations:** 1 Internal Medicine, California Institute of Behavioral Neurosciences & Psychology, Fairfield, USA; 2 Internal Medicine, Soroka University Medical Center, Beer Sheva, ISR; 3 Psychiatry and Neuroscience, California Institute of Behavioral Neurosciences & Psychology, Fairfield, USA; 4 Internal Medicine, California Institute of Behavioral Neurosciences & Psychology, Fairfield, USA, California, USA; 5 Otolaryngology, California Institute of Behavioral Neurosciences & Psychology, Fairfield, USA; 6 Surgical Oncology, California Institute of Behavioral Neurosciences & Psychology, Fairfield, USA

**Keywords:** sleep disorders, insomnia, sleepiness, restless leg syndrome, sleep apnea, peritoneal dialysis, hemodialysis, end-stage renal disease, chronic kidney disease

## Abstract

Chronic kidney disease (CKD) causes various complications that significantly impact a patient’s overall well-being and quality of life. Sleep disorders are a particularly common issue, especially in patients with advanced disease. This systematic review aims to explore the distinguishing features, prevalence rates, underlying causes, and associated factors related to the most frequent sleep disorders in these patients and present the latest treatment methods for them. It also investigates the link between CKD and sleep disorders and presents the results of the most common sleep disorders found in patients with CKD. Four major sleep disorders have been identified: sleep apnea, restless leg syndrome, excessive drowsiness, and insomnia. These sleep disorders have been discovered to be highly common in CKD patients and have a major influence on their quality of life and morbidity.

## Introduction and background

Chronic kidney disease (CKD) affects a significant portion of the global population, with an estimated prevalence of 10% worldwide, and it is estimated that over one million people will start dialysis for end-stage renal disease (ESRD) in the next decade [1]. The steady decline of kidney function over time that characterizes CKD is a degenerative disorder that negatively impacts overall health and quality of life.

Advanced CKD patients typically experience sleep difficulties, which impact a sizable majority of patients [2,3]. The frequency of sleep disorders can be as high as 80% when considering patients with ESRD receiving dialysis [4]. These sleep disorders have repeatedly been connected to a deterioration in general quality of life and an elevated risk of consequences.

As a result, treating and effectively managing sleep disorders in this group of patients is critical for improving patients’ well-being and minimizing potential negative consequences [5]. For those with severe CKD, a variety of variables contribute to the development of sleep disorder symptoms. These concerns include physiological changes associated with decreased kidney function, interruptions in sleep patterns induced by dialysis, poor sleep hygiene, and probable pharmaceutical adverse effects [6,7].

In this systematic review, we aim to explore the most prevalent types of sleep disorders found in patients with CKD. Specifically, we will focus on sleep apnea, restless leg syndrome (RLS), excessive sleepiness, and insomnia. We found those sleep disorders to be the most common in this patient population [8-11]. Our main goal is to provide a comprehensive understanding of the sleep-related challenges faced by individuals with CKD and their healthcare providers by exploring the characteristics, prevalence, associated factors, and potential interventions related to these sleep disorders.

## Review

Methods

This systematic review follows the Preferred Reporting Items for Systematic Reviews and Meta-Analyses (PRISMA) guidelines [12] to ensure rigorous methodology.

Search Strategy

The search was conducted using reputable sources, including PubMed, Google Scholar, and Embase; the last search date was April 23rd, 2023. An advanced medical subject headings (MeSH) systematic search strategy was employed to identify relevant studies within PubMed, using the advanced search with two main concepts as follows: End-stage kidney disease OR End-stage renal failure OR ("Chronic Kidney Diseases of Uncertain Etiology/etiology"[Majr] OR "Chronic Kidney Diseases of Uncertain Etiology/prevention and control"[Majr] OR "Chronic Kidney Diseases of Uncertain Etiology/rehabilitation"[Majr] OR "Chronic Kidney Diseases of Uncertain Etiology/therapy"[Majr] OR "Kidney Failure, Chronic/complications"[Majr] OR "Kidney Failure, Chronic/epidemiology"[Majr] OR "Kidney Failure, Chronic/prevention and control" [Majr] OR "Kidney Failure, Chronic/rehabilitation"[Majr] OR "Kidney Failure, Chronic /therapy"[Majr]) AND Sleep disorders OR insomnia OR sleepiness OR ("Sleep Initiation and Maintenance Disorders/drug therapy"[Majr] OR "Sleep Initiation and Maintenance Disorders/etiology"[Majr] OR "Sleep Initiation and Maintenance Disorders/prevention and control"[Majr] OR "Sleep Initiation and Maintenance Disorders /rehabilitation"[Majr] OR "Sleep Initiation and Maintenance Disorders/therapy"[Majr] OR "Restless Legs Syndrome/complications"[Majr] "Restless Legs Syndrome/etiology"[Majr] OR "Restless Legs Syndrome/therapy"[Majr] OR "Sleep Apnea Syndromes/complications "[Majr] OR "Sleep Apnea Syndromes/etiology"[Majr] OR "Sleep Apnea Syndromes/therapy "[Majr] OR "Disorders of Excessive Somnolence/complications"[Majr] OR "Disorders of Excessive Somnolence/drug therapy"[Majr] OR "Disorders of Excessive Somnolence /etiology"[Majr] OR "Disorders of Excessive Somnolence/rehabilitation"[Majr] OR "Disorders of Excessive Somnolence/therapy"[Majr]).

Eligibility Criteria

Studies were screened based on the types of studies: randomized controlled trials (RCTs), systematic reviews and meta-analyses, and observational studies. The inclusion criteria encompassed studies conducted on human adults, published in the last 10 years (2013 to 2023), and written in English. Conversely, non-English studies, studies with unavailable full texts or incomplete outcome data, studies involving pediatric populations, and studies published before 2013 were excluded. A detailed description of the inclusion and exclusion criteria is given in Table 1.

**Table 1:** Detailed inclusion and exclusion criteria

	Inclusion criteria	Exclusion criteria
1.	Papers from 2013 to 2023	Papers before 2013
2.	Papers published in the English language	Papers not published in the English language
3.	Papers focusing on the adult population (>18 years old)	Papers discussing the pediatric population (<18 years old)
4.	Observational studies, randomized controlled trials, systematic reviews, and meta-analyses	Case reports, letters, expert opinions, animal studies, grey literature, unpublished literature
5.	Papers relevant to the question	Papers irrelevant to the question
6.	Papers with full text and complete outcome data	Papers with unavailable full texts or incomplete outcome data

Analysis of Study Quality/Bias

We critically evaluated 19 selected studies for quality using standardized quality assessment tools, and 16 studies qualified as medium or high quality and were included in the review. The quality assessment of studies was performed using the Cochrane risk of bias tool for randomized controlled trials (RCTs), the New Castle Ottawa tool for observational studies, and the A Measurement Tool to Assess Systematic Reviews (AMSTAR) checklist for systematic reviews. The detailed overall scores and quality for each study selected are provided in Tables 2, 3.

**Table 2:** Summary of the AMSTAR tool for systematic reviews and meta-analyses AMSTAR: Assessment of Multiple Systematic Reviews; RoB: Risk of bias; PICO: Patient/population, intervention, comparison, outcome

AMSTAR criteria (yes, partial yes, no)	Studies				
	Rhee et al. (2020) [13]	Tan et al. (2022) [14]	So et al. (2020) [15]	Chen et al. (2022) [16]	Song et al. (2018) [17]
Did the research questions and inclusion criteria for the review include PICO components?	Yes	Yes	Yes	Yes	Yes
Did the report of the review contain an explicit statement that the review methods were established prior to the conduct of the review, and did the report justify any significant deviations from the protocol?	Yes	Yes	Yes	Yes	Yes
Did the review authors explain their selection of the study designs for inclusion in the review?	Yes	Yes	Yes	Yes	Yes
Did the review authors use a comprehensive literature search strategy?	Yes	Yes	Partial yes	Yes	Yes
Did the review authors perform study selection in duplicate?	Yes	Yes	Yes	Yes	Yes
Did the review authors perform data extraction in duplicate?	Yes	Yes	Yes	Yes	Partial yes
Did the review authors provide a list of excluded studies and justify the exclusions?	Yes	Yes	Yes	Yes	Yes
Did the review authors describe the included studies in adequate detail?	Yes	Yes	Partial yes	Yes	Yes
Did the review authors use a satisfactory technique for assessing the RoB in individual studies that were included in the review?	Yes	Yes	Yes	Yes	Yes
Did the review authors report on the funding sources for the studies included in the review?	Yes	Yes	No	Yes	No
If meta-analysis was performed, did the review authors use appropriate methods for the statistical combination of results?	Yes	Yes	Partial yes	Yes	Yes
If meta-analysis was performed, did the review authors assess the potential impact of RoB in individual studies on the results of the meta-analysis or other evidence synthesis?	Partial yes	Yes	Yes	Yes	Yes
Did the review authors account for RoB in individual studies when interpreting/discussing the results of the review?	Yes	Yes	Yes	Yes	Yes
Did the review authors provide a satisfactory explanation for and discussion of any heterogeneity observed in the results of the review?	Yes	Yes	Yes	Yes	Partial yes
Did the review authors provide a satisfactory explanation for and discussion of any heterogeneity observed in the results of the review?	Yes	Yes	Yes	Yes	Partial yes
Did the review authors report any potential sources of conflict of interest, including any funding they received for conducting the review?	Yes	No	Yes	Yes	Yes
Total score	15/16 (high quality)	14/16 (high quality)	13/16 (high quality)	16/16 (high quality)	13/16 (high quality)

**Table 3:** Summary of the Newcastle-Ottawa risk-of-bias tool for observational studies Quality check was done as per the Newcastle-Ottawa Scale (1, 0, N/A). N/A: Not applicable

Author & year of publication	Lyons et al. (2015) [18]	Castillo-Torres et al. (2014) [19]	Huang et al. (2018) [20]	Ogna et al. (2015) [21]	Lyons et al. 2015 [22]	Kahvecioglu et al. (2016) [23]	Lin et al. (2019) [24]	Tuohy et al. (2016) [25]
Selection								
Representativeness of the exposed cohort	1	1	1	1	1	1	1	1
Selection of the non-exposed cohort	1	1	1	1	1	1	1	1
Ascertainment of exposure	1	1	1	1	1	1	1	1
Demonstration that outcome of interest was not present at the start of the study	1	1	1	1	1	1	1	1
Comparability								
Study controls for most important factor (age)	1	1	1	1	1	1	1	1
Study controls for any additional factor(s)	1	0	1	1	1	1	1	1
Outcome								
Assessment of outcome	1	1	1	1	1	1	1	1
Was follow-up long enough for outcomes to occur?	1	1	1	1	1	1	N/A	1
Adequacy of follow-up of cohorts	1	0	0	0	0	0	N/A	1
Total	9/9	7/9	8/9	8/9	8/9	8/9	7/9	9/9
Quality	High	Medium	High	High	High	High	Medium	High

Results

**Figure 1:**
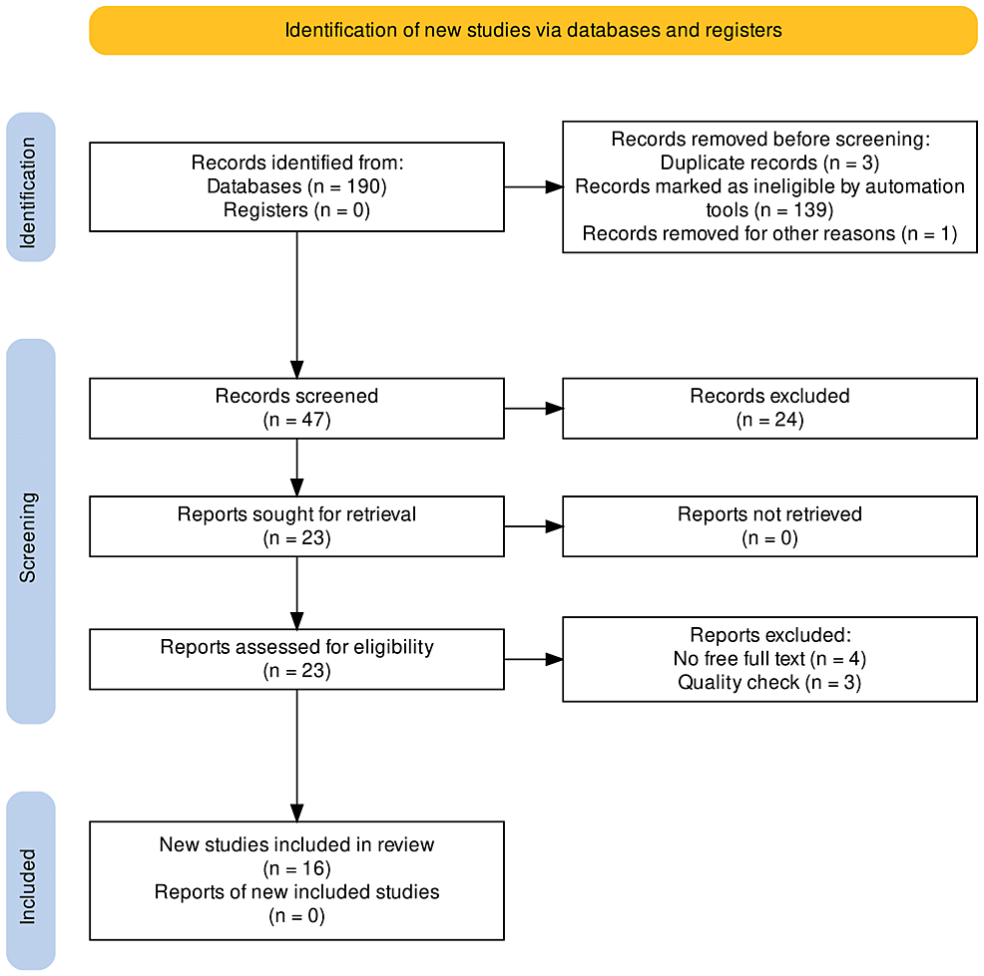
PRISMA Flow Diagram PRISMA: Preferred Reporting Items for Systematic Reviews and Meta-Analyses [12]

A total of 190 articles were identified in our initial search of PubMed, Google Scholar, and Embase databases. Out of them, 139 articles were discarded after applying relevant filters as per our eligibility criteria, and three duplicates were removed. Two individual investigators then screened the remaining 47 articles based on titles, abstracts, full-text, detailed inclusion-exclusion criteria, and quality/bias assessment. After the meticulous screening, we were left with 16 articles about our research question (Figure 1).

Out of the 16 included studies, there were eight observational studies, three RCTs, and five systematic reviews and meta-analyses. They included 2918 patients with different stages of CKD who reported a variety of sleep disorders.

Discussion

Sleep Apnea

Obstructive sleep apnea (OSA) and central sleep apnea (CSA) are two distinct sleep disorders that have different rates of prevalence in the general population. Recent data demonstrated that OSA affects 3% to 17% of people, whereas CSA affects less than 1% of the general population. However, in patients with ESRD, the prevalence of both central and obstructive sleep apnea is up to 50% to 60% [18].

Along with obesity, increased neck circumference, and metabolic syndrome, there are some ESRD-specific risk factors like fluid changes, airway edema, uremia, and decreased chemosensitivity that may contribute to the prevalence of sleep apnea in these patients [25]. Recent developments in the understanding of the pathophysiology of sleep apnea in ESRD have been made possible by the idea of nighttime rostral fluid shift [21].

The fact that both hemodialysis (HD) and peritoneal dialysis patients exhibit a greater prevalence of both sleep apnea disorders than the general population may suggest that the pathophysiology of sleep apnea is intimately connected to the development of kidney disease. Recent research has demonstrated a strong relationship between the rate of reduction in eGFR and the risk of developing OSA, with prevalence rates in patients undergoing HD reaching 56% and in patients with CKD ranging from 34% to 65% [20].

The link between sleep apnea and higher rates of overall mortality in ESRD patients has been well established [21]. Although sleep apnea has been linked to an increased risk of heart disease such as atrial fibrillation, the prevalence of sleep apnea does not seem to be connected with an increased risk of myocardial infarction or ischemic stroke [25].

Treating sleep apnea in patients with ESRD follows the same protocol as treating patients without this condition, but additional measures, such as switching the way kidney replacement therapy (KRT) regimens are administered, have been shown to help these individuals with their sleep apnea symptoms [22]. Nocturnal, or nighttime HD, has been found to significantly enhance sleep quality, lessen daytime sleepiness, and lessen the severity of sleep apnea, and has been shown to improve overall health outcomes for ESRD patients [10]. Furthermore, the reduction in fluid overload during HD and the decline in the obstructive apnea-hypopnea index were significantly correlated, even though HD by itself did not show a direct benefit on the severity of sleep apnea [21].

In addition to the adjustments made to the KRT regimen, another treatment option that addresses both kidney failure and related sleep apnea in patients with ESRD is kidney transplantation. A recent cross-sectional study suggested significant improvement in sleep apnea following successful kidney transplantation [23]. By recognizing the complex association between kidney disease and sleep apnea, healthcare providers can personalize interventions to address the specific needs of ESRD patients, leading to improved management and care outcomes.

Restless Leg Syndrome

Restless leg syndrome is a chronic sensory-motor disorder characterized by uncomfortable or abnormal sensations in the legs or arms, along with an irresistible urge to move the limbs. It is substantially more common in ESRD patients than it is in the general population. Due to sleep problems, insomnia, and even depressive symptoms, RLS has a profoundly negative impact on the quality of life of ESRD patients. Its symptoms are more prominent in patients during dialysis sessions, which makes resting uncomfortable [24,26].

Patients with ESRD are at risk of developing RLS due to several factors. Iron-deficiency anemia, which frequently coexists with anemia caused by chronic kidney failure in those patients, is one of them. Other risk factors include frequent and excessive alcohol consumption, being a woman, and type 2 diabetes [26,27].

The exact mechanisms causing RLS are not fully understood, but dopamine dysfunction in the central nervous system is thought to play a major role. Additionally, iron deficiency, commonly observed in ESRD patients as mentioned earlier, is considered a contributing factor to secondary RLS in this population. Female patients with ESRD also appear to have a higher prevalence, potentially due to higher estrogen levels in advanced kidney failure [19,27].

Diagnosing RLS is made primarily upon clinical presentation and involves recognizing the four main diagnostic criteria, such as the strong desire to move the extremities that is usually accompanied by abnormal sensations, worsening of symptoms at rest, partially or totally relieved with movement, and worsening of symptoms in the evening or at night [24].

Restless leg syndrome treatment options include nonpharmacologic and pharmacologic approaches. If RLS symptoms are mild or infrequent, nonpharmacologic interventions such as exercise training or intradialytic stretching may be useful [16,28]. Notably, recent studies have unveiled the efficacy of aerobic exercise as a highly effective management strategy for RLS among individuals undergoing chronic HD, leading to substantial improvements in symptom severity. Exercise training also demonstrated its potential to address other possible concomitant symptoms such as depression and fatigue, offering holistic benefits to HD patients [17,27]. A recent study showed that near-infrared light therapy has demonstrated promising results in alleviating the severity of RLS symptoms in HD patients [26]. Another possible technique is using cooler dialysate set at 35.5°C, which proved its efficiency in those patients by reducing RLS severity, fatigue, and cardiovascular mortality [16].

In terms of pharmacologic interventions for primary RLS, the recommended first-line treatments are non-ergot dopamine receptor agonists or alpha-2-delta calcium channel ligands. A recent study found that a transdermal patch containing rotigotine, a non-ergot dopamine receptor agonist, improved RLS symptoms in HD patients. This transdermal patch ensures continuous drug delivery and stable plasma levels throughout the day [28]. Gabapentin, an alpha-2-delta calcium channel ligand, is another very effective pharmacological treatment used to reduce RLS symptoms, especially if administered after dialysis sessions at a dosage of 100 to 300 mg [16].

Excessive Sleepiness

Excessive sleepiness, also known as hypersomnolence, refers to an increased tendency to fall asleep. It depicts the complex relationship between our circadian rhythms, or natural sleep-wake cycles, and our degree of general alertness [15]. Patients with ESRD on dialysis and those who had successful kidney transplants both reported sleepiness as a common symptom. In the CHOICE research (86%) and the LUCID trial (68%) of dialysis patients, excessive drowsiness was noted [27].

Patients with ESRD may experience sleepiness for a variety of reasons, including undiagnosed medical diseases, drug side effects, poor sleep hygiene, breathing problems during sleep, or primary central hypersomnia disorders such as narcolepsy [15]. Researchers also discovered that as people aged, their tendency to become sleepy decreased. Additionally, it was discovered that women were more likely than men to have extreme tiredness [27].

Patients with ESRD who are experiencing excessive sleepiness have some additional therapeutic choices available in addition to the standard therapies for the general population. Nocturnal HD is one of these options, which has the potential to reduce daytime sleepiness in ESRD patients with OSA [22].

Unfortunately, HD does have one significant drawback for ESRD patients. Melatonin production and blood levels are directly impacted, which is a hormone essential for sleep and controlling the circadian rhythm of the body. Patients receiving daily HD consequently see a decrease in the nocturnal surge of melatonin, which causes sleep disruptions. Melatonin administration was reported to improve the quality of sleep in dialysis patients in a recent randomized controlled experiment [15]. These results imply that melatonin administration might be an effective strategy for treating sleep issues in dialysis patients.

Insomnia

Insomnia, also known as sleeplessness, is a condition characterized by the difficulty of falling and staying asleep despite adequate sleep opportunities [15]. An estimated 50% to 75% of individuals with kidney failure and 8% to 36% of people with CKD in its initial stages report experiencing insomnia symptoms. Poor sleep and insomnia are particularly prevalent in the later stages of CKD due to uremia-related metabolic abnormalities, including refractory pruritus or RLS, which can worsen the course of CKD and increase mortality in dialysis patients [14].

Although several variables may play a major role in the onset of insomnia in individuals with CKD, the pathophysiologic processes causing it are not entirely understood. High blood orexin levels, low melatonin levels, and chronic inflammation brought on by uremia are thought to be key contributors to the etiology of insomnia [14]. The reason patients with ESRD experience insomnia may also be a result of other variables, such as pruritus, RLS, untreated sleep apnea, sadness, and anxiety, which are typical for most patients with CKD [13].

Clinically, patients with primary insomnia are distinguished by the fact that they struggle to get enough sleep even when they are provided with the opportunity, while patients with irregular sleep schedules manage to get sufficient sleep, although at inconvenient times. In addition, those with primary insomnia often spend extended periods awake in bed, resulting in fragmented sleep of four to five hours despite spending 10 or more hours in bed. On the other hand, a patient with an irregular sleep pattern, such as sleeping for four to five hours during dialysis and then getting three to four hours of sleep overnight, can still obtain eight hours of sleep within a 24-hour period. This difference is attributed to inadequate sleep hygiene [15].

The approach and management of insomnia in patients with CKD are similar to those in the general population, but a few additional measures may be considered. One of them, as mentioned earlier, is the timing of HD sessions, showing that evening dialysis is associated with improved sleep quality compared to daytime dialysis [14]. Additionally, kidney transplantation has been found to significantly reduce the prevalence of insomnia among patients with ESRD [23].

Limitations

While our systematic review provides valuable insights into sleep disorders in patients with CKD, there are a few limitations to consider. First, the included studies were limited to those published in English within the past 10 years, potentially excluding relevant research published in other languages or prior to 2013. Second, the review primarily focused on RLS, insomnia, sleep apnea, and excessive sleepiness, potentially overlooking other less common sleep disorders in patients with CKD. Lastly, the review relied on the quality and availability of the included studies, which could vary and potentially impact the overall findings and conclusions.

## Conclusions

Overall, this systematic review provides a thorough overview of the most common sleep disorders in patients with different stages of CKD, emphasizing the significance of personalized therapies for improved management and patient care outcomes. The findings highlighted the importance of healthcare providers recognizing and treating sleep disorders in patients with CKD since they have a major influence on quality of life and contribute to consequences. Future studies should concentrate on delving deeper into the mechanisms behind sleep disorders in patients with CKD and assessing specific therapies for these patients.
